# Comparison of Methods Utilizing Sex-Specific PRSs Derived From GWAS Summary Statistics

**DOI:** 10.3389/fgene.2022.892950

**Published:** 2022-07-08

**Authors:** Chi Zhang, Yixuan Ye, Hongyu Zhao

**Affiliations:** ^1^ Department of Biostatistics, Yale School of Public Health, New Haven, CT, United States; ^2^ Program of Computational Biology and Bioinformatics, Yale University, New Haven, CT, United States

**Keywords:** polygenic risk score, genome-wide association study, sex-specific, risk prediction, human genetics

## Abstract

The polygenic risk score (PRS) is calculated as the weighted sum of an individual’s genotypes and their estimated effect sizes, which is often used to estimate an individual’s genetic susceptibility to complex traits and disorders. It is well known that some complex human traits or disorders have sex differences in trait distributions, disease onset, progression, and treatment response, although the underlying mechanisms causing these sex differences remain largely unknown. PRSs for these traits are often based on Genome-Wide Association Studies (GWAS) data with both male and female samples included, ignoring sex differences. In this study, we present a benchmark study using both simulations with various combinations of genetic correlation and sample size ratios between sexes and real data to investigate whether combining sex-specific PRSs can outperform sex-agnostic PRSs on traits showing sex differences. We consider two types of PRS models in our study: single-population PRS models (PRScs, LDpred2) and multiple-population PRS models (PRScsx). For each trait or disorder, the candidate PRSs were calculated based on sex-specific GWAS data and sex-agnostic GWAS data. The simulation results show that applying LDpred2 or PRScsx to sex-specific GWAS data and then combining sex-specific PRSs leads to the highest prediction accuracy when the genetic correlation between sexes is low and the sample sizes for both sexes are balanced and large. Otherwise, the PRS generated by applying LDpred2 or PRScs to sex-agnostic GWAS data is more appropriate. If the sample sizes between sexes are not too small and very unbalanced, combining LDpred2-based sex-specific PRSs to predict on the sex with a larger sample size and combining PRScsx-based sex-specific PRSs to predict on the sex with a smaller size are the preferred strategies. For real data, we considered 19 traits from Genetic Investigation of ANthropometric Traits (GIANT) consortium studies and UK Biobank with both sex-specific GWAS data and sex-agnostic GWAS data. We found that for waist-to-hip ratio (WHR) related traits, accounting for sex differences and incorporating information from the opposite sex could help improve PRS prediction accuracy. Taken together, our findings in this study provide guidance on how to calculate the best PRS for sex-differentiated traits or disorders, especially as the sample size of GWASs grows in the future.

## Introduction

Many traits or complex diseases result from the combined influence of many genetic variants and other risk factors. The polygenic risk score (PRS) is a measure of an individual’s genetic susceptibility to a trait or complex disorder. It is calculated as the weighted sum of an individual’s genotypes and their estimated effect sizes. The PRS is developed to use genetic information to predict complex disorders and can also be informative to assess genetic overlap between traits (Choi et al., 2020). Because most individual variants only have weak effects on disease risk (Lewis and Vassos, 2020), the PRS is often calculated over a large number of single nucleotide polymorphisms (SNPs) to improve prediction accuracy. Ideally, the variability of phenotype explained by the PRS in the testing samples should be close to the heritability explained by the SNPs if the effect sizes of SNPs are accurate. However, because of uncertainties in the effect size estimation and inherent discrepancies between populations, the predictive power of PRSs is often significantly lower than that of SNP heritability (Choi et al., 2020). Aside from the simple PRS prediction method [e.g., P + T (Euesden et al., 2015)], a number of Bayesian methods have been developed to improve effect size estimation *via* different prior distribution specifications, leading to better prediction accuracy. In this article, we focus on three such PRS models, including PRScs (Ge et al., 2019), LDpred2 (Priv é et al., 2021), and PRScsx (Ruan et al., 2021) that have been shown to be among the best PRS methods (Ge et al., 2019; Privé et al., 2021; Ruan et al., 2021). Two of these methods, PRScs and LDpred2 (Ge et al., 2019; Priv é et al., 2021), are single-population PRS methods, whereas PRScsx (Ruan et al., 2021) is a multiple-population PRS method that was developed for cross-population predictions. The PRScsx framework can also be used for cross-sex predictions when females and males are considered separate populations.

In the applications of the PRS to different traits and disorders, the input is the summary statistics from genome-wide association studies (GWAS), which often combines female and male samples with the implicit assumption that the effect sizes are the same between the two sexes. However, many investigations have suggested sex differences in traits and in the risk of developing complex disorders in recent years. For example, in the context of Alzheimer’s disease, females acquire verbal memory impairments later but exhibit decline faster than in males (Caldwell et al., 2017). There are obvious differences between males and females in anthropometric traits (Traglia et al., 2016). Another example are the blood cell traits, which reveal well-known sex differences when researchers explore the role of sex in genetic effects on these traits (Khramtsova et al., 2018). It has been shown that PRSs for around half of the blood cell traits result in different levels of stratification between men and women (Xu et al., 2022). Furthermore, when a sex-stratified GWAS meta-analysis for lipid levels was performed to evaluate the underlying biological pathways and mechanisms of blood lipid levels (Kanoni et al., 2021), it was discovered that three to five percent of autosomal lipid-associated loci had sex-biased effects and that many of these sex-biased autosomal lipid loci have pleiotropic associations with sex hormones (Kanoni et al., 2021). Some of these sex differences are thought to be explained by genotype-by-sex interactions (GxS). A biobank-scaled study of about 530 phenotypes gave insights into both the scope and mechanism of GxS, revealing tiny but broad sex differences in genomic architecture across phenotypes (Bernabéu et al., 2021). It also suggests that sex-agnostic studies may be overlooking trait-associated loci and that utilizing sex-specific SNPs may enhance prediction (Bernabéu et al., 2021).

To address sex-specific genetic effects, sex-specific PRSs have been developed and evaluated (Roberts et al., 2019; Fan et al., 2020; Flynn et al., 2020). However, there has not been a detailed study on whether and how GWAS results for females and males should be considered in producing PRSs to achieve better prediction accuracy for both sexes. In this study, we perform comprehensive simulations and real data analysis to assess the performance of three GWAS-based PRS methods, namely PRScs, PRScsx, and LDpred2, with different strategies to generate PRSs. In simulations, the phenotypes were generated using UK Biobank genotype data (Bycroft et al., 2018), and we considered both balanced and unbalanced sample sizes of females and males. We further compared the performance of these approaches under various settings of genetic correlation and heritability. In real data analysis, we evaluated these models using sex-specific and sex-agnostic GWASs from the Genetic Investigation of ANthropometric Traits (GIANT) collaboration (Randall et al., 2013). We also generated sex-specific and sex-agnostic GWASs from the UK Biobank (Bycroft et al., 2018) using BOLT-LMM (Loh et al., 2015) to do further real data analysis. Our results shed light on the utility of sex-specific PRSs, and we offer recommendations for their use in a variety of contexts.

## Results

### Overview of Polygenic Risk Score Construction

**FIGURE 1:**
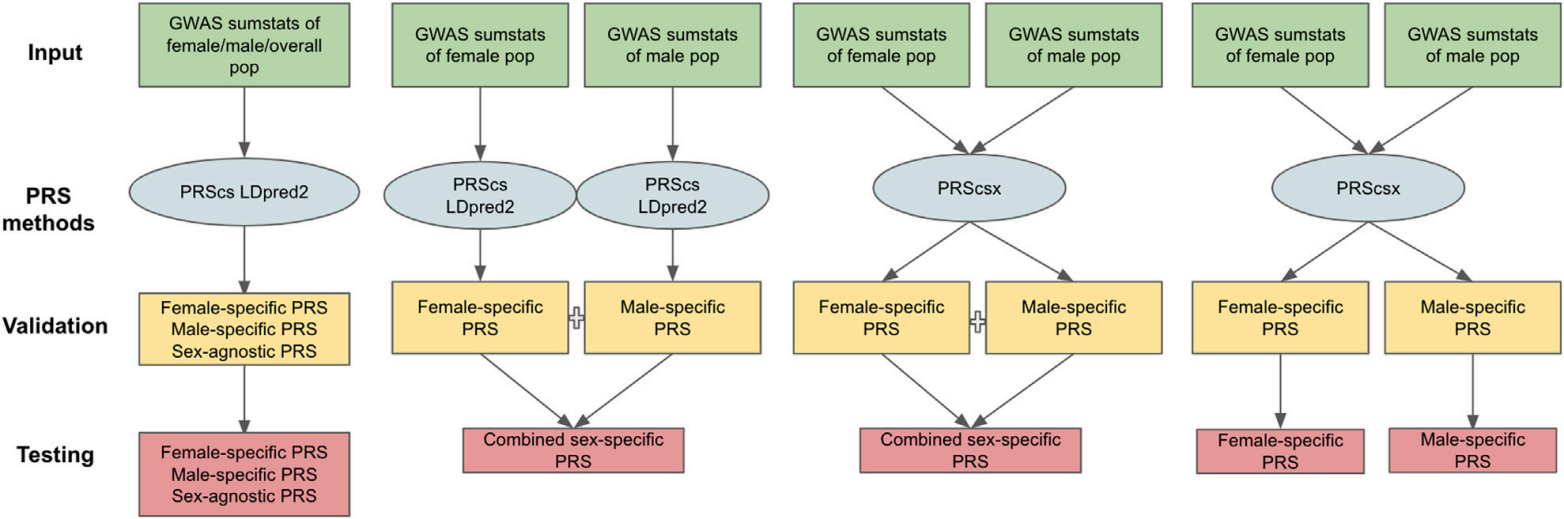
Overview of the 11 PRSs considered. The inputs are female-specific GWAS summary statistics, male-specific GWAS summary statistics, and sex-agnostic GWAS summary statistics; PRS methods include PRScs, LDpred2, and PRScsx; validation samples are used to tune hyperparameters of PRSs and find the optimal weights of sex-specific PRSs; testing samples are to assess prediction accuracy.

The PRS methods considered in our work can be classified into two categories: those designed for one population [PRScs (Ge et al., 2019) and LDpred2 (Priv é et al., 2021)] and for multiple populations [PRScsx (Ruan et al., 2021)]. One-population methods only take one set of GWAS summary statistics as input, whereas multiple-population models can use GWAS summary statistics from several populations. In this study, we treat males and females as separate populations and generate three types of GWAS summary statistics: 1) female-specific GWAS summary statistics based on female samples only; 2) male-specific GWAS summary statistics based on male samples only; and 3) sex-agnostic GWAS summary statistics considering both female and male samples. Applying PRScs and LDpred2 to the sex-agnostic GWAS summary statistics generated sex-agnostic PRScs and sex-agnostic LDpred2 scores. Sex-specific PRScs and sex-specific LDpred2 were produced when we applied PRScs and LDpred2 to respective sex-specific GWAS summary statistics. Since PRScsx calculates PRS for each population, when we input both female- and male-specific GWAS summary statistics, we could obtain female- and male-specific PRScsx simultaneously. Based on sex-specific PRSs, we can generate combined sex-specific PRSs (PRScs-mult, LDpred2-mult, and PRScsx-mult). They represent the optimal linear combination of sex-specific PRSs whose weights are learned on the validation data set. Thus, in total, we have 11 different PRSs: female-specific PRScs (PRScs-f), male-specific PRScs (PRScs-m), sex-agnostic PRScs (PRScs-all), combined sex-specific PRScs (PRScs-mult), female-specific LDpred2 (LDpred2-f), male-specific LDpred2 (LDpred2-m), sex-agnostic LDpred2 (LDpred2-all), combined sex-specific LDpred2 (LDpred2-mult), female-specific PRScsx (PRScsx-f), male-specific PRScsx (PRScsx-m), and combined sex-specific PRScsx (PRScsx-mult). The workflow for each PRS’s construction is shown in Figure 1.

### Simulation

We first conducted simulations to evaluate the prediction performance of sex-agnostic PRSs, sex-specific PRSs, and combined sex-specific PRSs derived from various PRS methods. We simulated individual-level genotypes of the white British population from the UK Biobank data for HapMap3 variants whose minor allele frequencies are larger than 1% (1,195,561 SNPs). We chose 1 KG Phase 3 European samples as our reference panel for all PRS methods. With balanced female and male sample sizes, we considered three sample sizes (females/males: 5,000/5,000; 25,000/25,000; 100,000/100,000) as the training data set to construct sex-specific and sex-agnostic GWAS summary statistics. We also conducted a simulation with unbalanced heritability between sexes when the sample sizes were 25,000 females and 25,000 males. In the unbalanced sample size settings, we considered three scenarios: females/males: 2,500/12,500, females/males: 10,000/50,000, and females/males: 50,000/10,000. We simulated 5,000 females and 5,000 males to form the validation data set, and another 5,000 females and 5,000 males as the testing data set. There was no overlap among the training, validation, and testing data sets. Among all the SNPs, we randomly sampled ∼0.1% HapMap3 variants (1,200 SNPs) as causal variants for each sex and assumed that the causal variants were shared across sexes and their effect sizes followed a bivariate normal distribution with genetic correlation (*rg*) set to 1, 0.8, and 0.5, respectively. We also considered the situation when only a subset of causal variants was shared across sexes. We conducted a simulation with 80% of causal variants shared across sexes and their effect sizes following a bivariate normal distribution with genetic correlation (*rg*) set to 0.8 and 0.5. The squared correlation (R-squared) between simulated traits and predicted traits was calculated to evaluate the performance of the PRSs, and each simulation setting was repeated ten times.

#### Balanced Sample Size

The simulated traits of both males and females were generated by the sum of all causal markers, weighted by the true effect sizes with the heritability of 0.3. We applied PRScs, PRScsx, and LDpred2 to sex-specific GWAS summary statistics generated from 5,000 female/male samples and sex-agnostic GWAS summary statistics generated from 10,000 samples. Sex-agnostic LDpred2 (LDpred2-all) showed the best prediction performance for both sexes and in all genetic correlation settings (Supplementary Figure S1). When the sample size was increased to 25,000 females and 25,000 males, combined sex-specific LDpred2 (LDpred2-mult) had a higher prediction accuracy than the other PRSs when the genetic correlation between the sexes was 0.5. However, when the genetic correlation was 1 or 0.8, the sex-agnostic LDpred2 (LDpred2-all) consistently outperformed the other PRSs (Supplementary Figure S2; Table 1). When the sample size was increased to 100,000 females and 100,000 males, sex-agnostic PRScs (PRScs-all) performed the best when the genetic correlation was 1, especially better than sex-agnostic LDpred2 (LDpred2-all), which may indicate that PRScs performs better than LDpred2 when the sample size is very large. However, when the genetic correlation decreased to 0.5 and 0.8, combined sex-specific PRScsx (PRScsx-mult) outperformed the other methods, and it had a similar performance to the corresponding sex-specific PRScsx (Supplementary Figure S3; Table 1). In this case, combined sex-specific PRScsx (PRScsx-mult) started to perform better than combined sex-specific LDpred2 (LDpred2-mult) which may further indicate methods using continuous shrinkage prior (PRScs, PRScsx) may have better prediction performance than LDpred2 when the sample size is very large. Furthermore, in all simulations, the combined sex-specific PRScsx (PRScsx-mult) outperformed the combined sex-specific PRScs (PRScs-mult) and the sex-specific PRScsx outperformed the sex-specific PRScs, indicating that integrating GWAS summary statistics from multiple populations could improve PRS prediction. We also conducted a simulation when the proportion of causal SNPs shared across sexes was 80% (Supplementary Figure S4; Table 1). Combined sex-specific LDpred2 (LDpred2-mult) performed the best when the genetic correlation was 0.5 which presented a consistent finding when we assumed that all the causal SNPs were shared between females and males.

**TABLE 1:** The median and standard deviation of R-squared for each method in different simulation settings. We only show the outcomes of simulated settings that benefit from sex-specific PRSs. Because the prediction performances on females and males are consistent when sample sizes and heritability are both balanced across sexes, we only report the prediction accuracy on females in these cases. ***Bold and italics*** indicate the best results in each simulation setting, and the second-best results are presented only in **bold**.

Sample Sizes (females/males)		**25,000/25,000**		**100,000/100,000**		**10,000/50,000**		**25,000/25,000**
Heritability (females/males)		**0.3/0.3**						**0.1/0.5**
Proportion of shared causal SNPs		**0.8**	**1**	**1**		**1**		**1**
Genetic correlation		**0.5**		**0.8**	**0.5**	**0.5**		**0.5**
Testing samples		Females		Females		Females	Males	Males
Combined sex	LDpred2-mult	***0.132* (*0.030*)**	***0.141* (*0.004*)**	0.223 (0.011)	0.224 (0.013)	0.078 (0.005)	***0.191 (0.010)***	***0.282* (*0.008*)**
	PRScs-mult	0.102 (0.010)	0.106 (0.007)	0.224 (0.014)	0.219 (0.012)	0.062 (0.008)	0.162 (0.008)	0.246 (0.010)
	PRScsx-mult	0.121 (0.010)	0.128 (0.007)	***0.235* (*0.014*)**	***0.239* (*0.013*)**	***0.094* (*0.005*)**	0.172 (0.009)	0.256 (0.013)
Female-specific	LDpred2-f	**0.130 (0.010)**	**0.132 (0.005)**	0.217 (0.011)	0.225 (0.013)	0.049 (0.007)	0.012 (0.003)	0.016 (0.004)
	PRScs-f	0.094 (0.009)	0.099 (0.007)	0.219 (0.013)	0.217 (0.012)	0.031 (0.005)	0.007 (0.003)	0.010 (0.003)
	PRScsx-f	0.121 (0.010)	0.128 (0.007)	**0.234 (0.014)**	**0.238 (0.013)**	**0.091 (0.005)**	0.058 (0.008)	0.085 (0.014)
Male-specific	LDpred2-m	0.030 (0.010)	0.029 (0.007)	0.133 (0.010)	0.052 (0.008)	0.045 (0.005)	***0.191* (*0.010*)**	**0.280 (0.008)**
	PRScs-m	0.025 (0.006)	0.026 (0.006)	0.130 (0.011)	0.050 (0.006)	0.040 (0.006)	0.162 (0.009)	0.244 (0.010)
	PRScsx-m	0.049 (0.009)	0.052 (0.007)	0.168 (0.012)	0.071 (0.011)	0.052 (0.007)	0.171 (0.009)	0.256 (0.013)
Sex-agnostic	LDpred2-all	2 0.116 (0.009)	0.122 (0.009)	0.197 (0.009)	0.172 (0.015)	0.073 (0.006)	0.187 (0.010)	0.234 (0.015)
	PRScs-all	0.093 (0.010)	0.103 (0.009)	0.210 (0.014)	0.173 (0.011)	0.067 (0.010)	0.163 (0.007)	0.189 (0.013)

Bernabéu et al. (2021) has found that the estimated heritability differs between sexes for the same trait, which suggests a difference in the proportion of a trait’s variance accounted for by the genotypes, and hence a possible sex difference in the trait’s underlying genetic architecture. The differences in heritability between the sexes are statistically significant for some traits, for example, body mass–related traits (Bernabéu et al., 2021). Thus, we also conducted simulations where the heritability differed between sexes. We chose 25,000 females and 25,000 males to derive GWAS summary statistics. The causal variants explained 10% of the phenotypic variation in females, while in males, the causal variants explained 50%. As shown in Supplementary Figure S5 and Table 1, when the genetic correlation was 0.5 and 0.8, sex-agnostic LDpred2 (LDpred2-all) outperformed the female predictions, which had a lower heritability, while for the male predictions, the combined sex-specific LDpred2 (LDpred2-mult) and male-specific LDpred2 (LDpred2-m) were comparable with each other and performed better than the other PRSs. When the genetic correlation was 1, sex-agnostic LDpred2 (LDpred2-all) and combined sex-specific LDpred2 (LDpred2-mult) were comparable and performed best on both females and males. Compared with the simulation results with the same heritability and the same sample size between sexes, it is more difficult to utilize the information of sex difference in PRS prediction for the sex with a lower heritability.

#### Unbalanced Sample Sizes

There are situations when the sample sizes are unbalanced between males and females. In the UK Biobank (UKB) data set, for example, there are more females than males. For the Million Veteran Program, males account for 92% of the study subjects (Program et al., 2019). Therefore, we did simulations to assess whether sex-specific PRSs or combined sex-specific PRSs (LDpred2-mult, PRScs-mult, and PRScsx-mult) can improve cross-sex polygenic prediction when the GWAS sample sizes are unbalanced. We set the heritability of females and males in this unbalanced sample size simulation to be 0.3. The ratio of female sample sizes to male sample sizes was first set to 1:5. First, when we generated 2,500 females and 12,500 males as the training data, the sex-agnostic LDpred2 (LDpred2-all) improved prediction accuracy over all the other PRSs on both sexes and in all genetic correlation settings (Supplementary Figure S6). When the training sample sizes were increased to 10,000 females and 50,000 males, in females, where the sample size was smaller for deriving female-specific GWAS summary statistics, combined sex-specific PRScsx (PRScsx-mult) had the best performance when the genetic correlation was 0.5 and was comparable with female-specific PRScsx (PRScsx-f). On the other hand, for males, the combined sex-specific LDpred2 (LDpred2-mult) performed better when genetic correlation was 0.5, and male-specific LDpred2 (LDpred2-m) and sex-agnostic LDpred2 (LDpred2-all) were comparable with it. When the genetic correlation was 1 or 0.8, sex-agnostic LDpred2 (LDpred2-all) remained the best performer among all the methods (Supplementary Figure S7; Table 1). We also swapped the sample size magnitudes for males and females to perform a simulation with 50,000 females and 10,000 males, and the results in Supplementary Figure S8 shows consistent results from the simulation with more males than females.

### Applications to Summary Statistics From Genetic Investigation of Anthropometric Traits

We obtained sex-specific GWAS summary statistics and sex-agnostic GWAS summary statistics for 12 traits (WHR, WHRadjBMI, HIP, HIPadjBMI, WC, WCadjBMI, BMI.SNPadjPA, BMI.SNPadjSMK, WHRadjBMI.SNPadjPA, WHRadjBMI.SNPadjSMK, WCadjBMI.SNPadjPA, and WCadjBMI.SNPadjSMK) from the GIANT study (Shungin et al., 2015). For all these traits, there were more females than males. We applied LDSC (Bulik-Sullivan et al., 2015) to calculate the genetic correlation between females and males and their respective heritability, with the results summarized in Supplementary Table S1.

**FIGURE 2:**
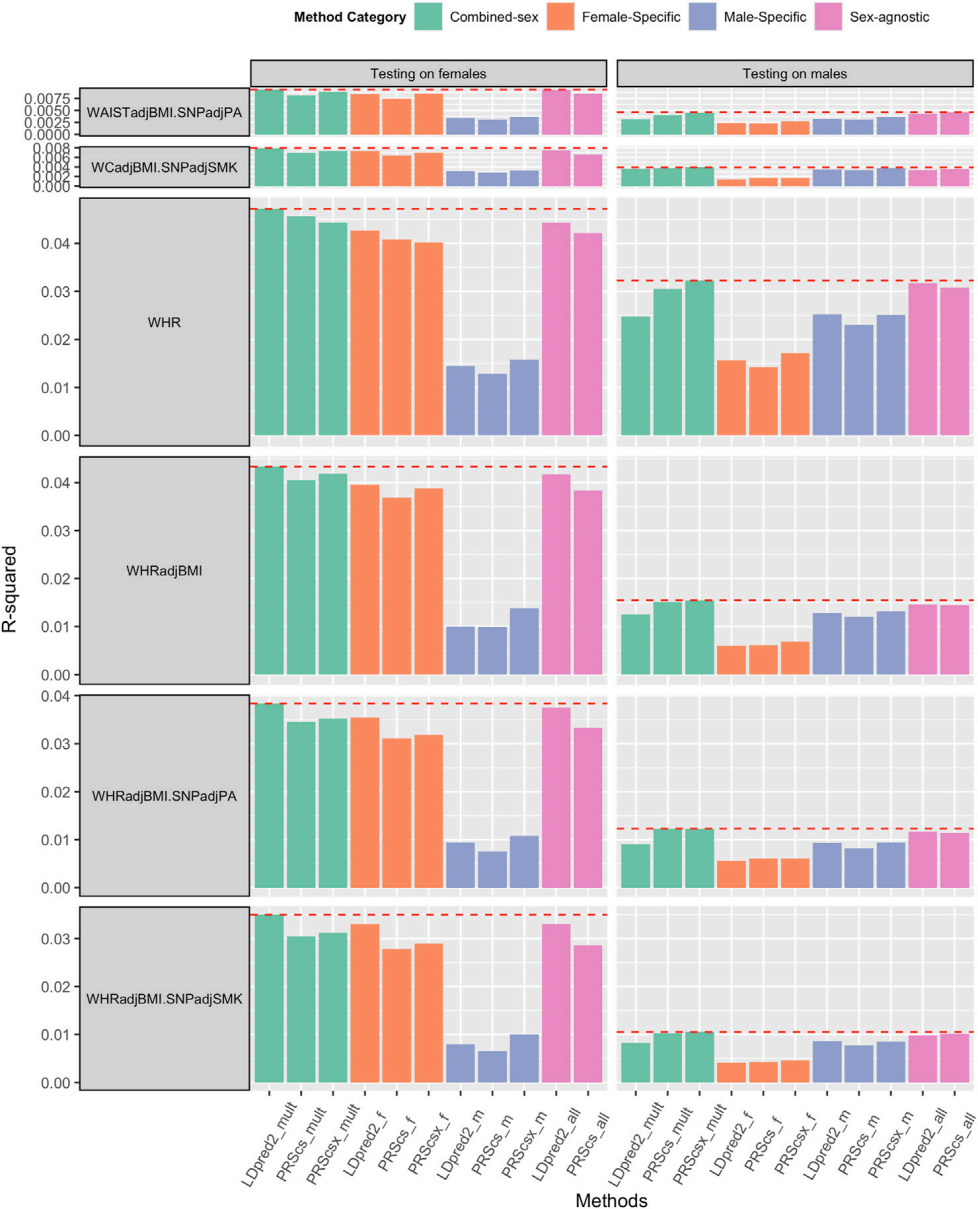
Comparisons of PRSs on WHR and WHRadjBMI, WHRadjBMI.SNPadjPA and WHRadjBMI.SNPadjSMK, and WCadjBMI.SNPadjPA and WCadjBMI.SNPadjSMK (WHR: waist and hip ratio; WHRadjBMI: WHR adjusted by BMI; WHRadjBMI.SNPadjPA: with physical activity level as a covariate; WHRadjBMI.SNPadjSMK: with smoking status as a covariate; WCadjBMI.SNPadjPA: with physical activity level as a covariate; WCadjBMI.SNPadjSMK: with smoking status as a covariate). **Female-Specific:** Using female-specific GWAS summary statistics as input. **Male-Specific:** using male-specific GWAS summary statistics as input. **Sex-agnostic:** using sex-agnostic GWAS summary statistics as input. **Combined-sex:** the combination of female-specific PRS and male-specific PRS.

We applied sex-agnostic PRS methods, sex-specific PRS methods, and combined sex-specific PRS methods to the sex-specific GWAS summary statistics and sex-agnostic GWAS summary statistics of each trait. We first investigated the PRS performance of WHR, WHRadjBMI, HIP, HIPadjBMI, WC, and WCadjBMI. Among these six traits, WHR and WHRadjBMI showed the benefit of sex-specific PRS and combined sex-specific PRS on both sexes (Figure 2). For female predictions, combined sex-specific LDpred2 (LDpred2-mult) had the best performance, which was much better than female-specific LDpred2 (LDpred2-f), which suggests the contribution of male-specific LDpred2. For male predictions, combined sex-specific PRScsx (PRScsx-mult) outperformed others, which suggests the ability of PRScsx to borrow information from the sex with a larger sample size. As shown in Supplementary Table S1, the genetic correlations of WHR and WHRadjBMI between females and males were 0.708 (SE: 0.021) and 0.664 (SE: 0.028), respectively, which are relatively low. These results are consistent with simulations for the imbalanced sample size setting that sex-specific or combined sex-specific methods could improve the prediction accuracy when the genetic correlation is low. However, for the other four traits, sex-agnostic LDpred2 (LDpred2-all) outperformed other PRSs, except for HIPadjBMI and WCadjBMI where LDpred2-mult performed the best on females (Supplementary Figures S9, S10).

We also obtained sex-specific GWAS summary statistics and sex-agnostic GWAS summary statistics of BMI, WHRadjBMI, and WCadjBMI after adding physical activity levels (BMI.SNPadjPA, WHRadjBMI.SNPadjPA, and WCadjBMI.SNPadjPA) or smoking status (BMI.SNPadjSMK, WHRadjBMI.SNPadjSMK, and WCadjBMI.SNPadjSMK) as covariant. Sex-agnostic LDpred2 (LDpred2-all) still outperformed other methods when predicting BMI for both females and males (Supplementary Figure S11). As for WHRadjBMI.SNPadjPA and WHRadjBMI.SNPadjSMK, combined sex-specific LDpred2 (LDpred2-mult) had the best prediction accuracy on females, and both combined sex-specific PRScs (PRScs-mult) and combined sex-specific PRScsx (PRScsx-mult) had the best performance for males (Figure 2). As for WCadjBMI.SNPadjPA and WCadjBMI.SNPadjSMK, when predicting for females, combined sex-specific LDpred2 (LDpred2-mult) outperformed others. When predicting for males, sex-agnostic LDpred2 (LDpred2-all) offered the best results for WCadjBMI.SNPadjPA and combined sex-specific PRScsx outperformed others for WCadjBMI.SNPadjSMK (Figure 2).

### Applications to Summary Statistics From UK Biobank

Based on prior knowledge (Han et al., 2021), we studied seven traits related to body fat mass/distribution using the UK Biobank samples. We first selected about 100,000 females and 120,000 males of white British ancestry as our training data set to generate sex-specific and sex-agnostic GWAS summary statistics using BOLT-LMM (Loh et al., 2015). The remaining UK Biobank white British samples were used to construct the validation and testing data sets. There were about 80,000 samples for validation (female/male: 39752/37122) and testing (female/male: 43644/36670), respectively. We applied LDSC (Bulik-Sullivan et al., 2015) to calculate the heritability and genetic correlation between females and males. The results are summarized in Supplementary Table S2. We can see that for all these traits, sex-agnostic LDpred2 (LDpred2-all) consistently outperformed the other PRS methods. Even though combined sex-specific LDpred2 was comparable with LDpred2-all in females, it underperformed when compared to LDpred2-all on these traits (Supplementary Figures S12, S13).

## Materials and Methods

### Study Population and Quality Control of Genotype Data

The sex-specific and sex-agnostic GWAS summary statistics were obtained from the Genetic Investigation of ANthropometric Traits (GIANT) collaboration (Randall et al., 2013). GIANT is an international project that uses meta-analysis of GWAS data and other large-scale genetic data sets to uncover genetic loci that control human body size and shape, such as height and obesity measures. So far, the GIANT group has discovered common genetic variations at hundreds of loci related to anthropometric traits (Shungin et al., 2015).

We also used the UK Biobank data to generate sex-specific and sex-agnostic GWAS summary statistics for several traits. The UK Biobank (UKB) (Bycroft et al., 2018) is a major prospective study that was designed to serve as a resource for research into complex traits and diseases in middle-aged adults. The study protocol, study design information, and data access are all available online (UK Biobank Analysis Team, 2011). Between 2006 and 2010, a total of 502,618 participants aged 40–69 years were recruited from 22 assessment centers across the United Kingdom. In our simulations, we used the imputed genotype data from UKB. We chose genetically unrelated participants of white British ancestry for our study. Ancestry is determined by a mix of self-reported ancestry and genetically confirmed ancestry determined through principal component analysis of the individuals’ genomes. Exclusion criteria included a lack of genetic data (sufficient DNA could not be extracted from the blood samples of ∼3% of participants), discordance between reported and genotype inferred sex, poor heterozygosity or missingness, sex chromosome aneuploidy, withdrawal of informed consent, and individuals with at least one relative. There were 380,978 samples left. Then, we divided these samples into three data sets that did not overlap with each other: training data, validation data, and testing data. In real data analysis, we chose 223,790 (female/male: 121,808/101,982) unrelated participants of white British ancestry who were in the first 60 batches as the training data set and 80,314 individuals (female/male: 43,644/36,670) from batches 61 to 85 were used as the testing data set. The other 76,874 individuals (female/male: 39,752/37,122) were treated as the validation data set. The exact sample sizes were varied when analyzing each trait because we excluded the missing values of that trait. We restricted the analysis to autosomal variants with a genotype missing rate of less than 0.05, an imputation quality score more than 0.3, a Hardy–Weinberg equilibrium *p*-value greater than 1e-6, and a minor allele frequency (MAF) greater than 0.05. We also eliminated all strand-ambiguous SNPs. To obtain sex-specific GWAS and sex-agnostic GWAS summary statistics, we used Bolt-LMM (Loh et al., 2015) on the training data set to estimate the marginal effect sizes of genetic variants. The sex-specific GWAS summary data were adjusted for age, age2, and the first 20 principal components. And the sex-agnostic GWAS summary data were correlated by sex, age, age2, age*sex, age*sex2, and the first 20 principal components.

### Polygenic Risk Score Models

**LDpred2** is a new version of LDpred with a larger window size of 3 cM. It could better handle numerical errors when working with exponentials. With an efficient parallel implementation in C++ and restrictions to HapMap3 variants, LDpred2 could run with a larger hyper-parameter search space than LDpred but with a shorter time. There are two extensions of LDpred2: 1) sparsity option in LDpred2-grid which provides models that truly encourage sparsity and 2) LDpred2-auto which automatically estimates values for hyper-parameters *p* and heritability. We tested a grid of hyper-parameters with *p* from a sequence of 17 values from 10e−5 to 1 on a log scale; *h*2 within {0.7,1,1.4} times heritability estimates from LDSC and their sparsity option.

**PRS-CS** is also a Bayesian method that calculates the posterior mean effect size of each variant based on GWAS summary statistics and linkage disequilibrium (LD). It employs a continuous shrinkage prior to SNP effect sizes, with one hyper-parameter, the global shrinkage parameter. This hyper-parameter represents the genetic architecture’s overall sparsity. The 1 KG LD reference panel used in this study was pre-calculated and built for HapMap3 variants with MAF >0.01. Though PRScs could automatically estimate the parameter, we use the version that tunes the parameter and the global shrinkage parameter is range from 1e-8 to 1e-6, 1e-4, 1e-2, to 1.

**PRS-CSx** is an extension of PRS-CS which enables the integration of GWAS summary statistics from multiple populations to improve cross-population prediction performance. We also use the version that tunes the parameter and the global shrinkage parameter that ranges from 1e-8 to 1e-6, 1e-4, 1e-2, to 1.

### Sex-specific Polygenic Risk Score and Combined Sex-specific Polygenic Risk Score

In all the PRS analyses, we applied PRS methods to sex-specific and sex-agnostic GWAS summary statistics to estimate the effect sizes of genetic variants. Then, the validation data set, with individual-level genotypes and phenotypes, was used to tune hyperparameters for different PRS methods. After we selected the best PRS for each method using the validation data set, we used the testing data set to evaluate their prediction accuracy and computed performance metrics which is the square of correlation for quantity phenotypes. For all the PRS methods, we used the 1000 Genomes Project (1 KG) Phase 3 super-population samples as the LD reference panels (Auton et al., 2015).

The PRS models in this work can be broadly grouped into two categories: the single population methods (LDpred2 and PRScs), which train PRS using one set of GWAS summary statistics, and the multi-population method (PRScsx), which uses GWAS summary statistics from several populations. First, sex-agnostic (GWAS created using both female and male samples) and sex-specific GWAS summary statistics (GWAS generated using either female or male sample) are derived from the training samples. We obtained female-specific PRScs/LDpred2, male-specific PRScs/LDpred2, and sex-agnostic PRScs/LDpred2 by applying PRScs and LDpred2 to these three sets of GWAS summary statistics. The female-specific PRScsx and male-specific PRScsx are generated by inputting both female-specific and male-specific GWAS summary statistics to PRScsx. We tuned sex-specific PRSs and sex-agnostic PRSs on the validation samples whose sex was the same as that of the testing data set which we were going to predict on. The combined sex-specific PRSs are created by linearly combining sex-specific PRSs on the target validation data set. The hyper-parameters and weights of each sex-specific PRS are validated simultaneously.

## Discussion

In this article, we have presented a comprehensive study to investigate what would be the most effective way to develop PRS that can adequately address sex differences in genetic associations for traits/disorders with demonstrated sex differences, such as different heritability and/or low genetic correlation between sexes. We considered sex-agnostic PRS, sex-specific PRS, and combined sex-specific PRS that are built from PRScs, LDpred2, and PRScsx. We have shown, *via* simulation studies, that when the sample sizes are small, even though there is a relatively small genetic correlation between sexes, the increase in sample size contributes more than sex differences in PRS prediction. With an increase in sample size, the combined sex-specific PRSs gradually improve their relative performance and the majority part of the information for combined sex-specific PRSs is from the corresponding sex-specific PRSs. When the sample sizes are large enough, PRScs and PRScsx provide better performance than LDpred2. Besides, combined sex-specific PRScsx always performs better than combined sex-specific PRScs, which indicates the benefits of jointly modeling multiple GWAS summary statistics. When there is a difference in heritability between the sexes, combined sex-specific PRSs can be an option for the sex with higher heritability. In unbalanced sample size simulations, we found that female-specific PRScsx consistently outperformed other female-specific PRSs in both sexes, where there were fewer females than males. This could imply that sex-specific PRS of the smaller sample size could benefit from information from the opposite sex with a larger sample size by PRScsx. When the sample size is unbalanced between sexes, the combined sex-specific PRScsx may be a good candidate PRS to predict on the sex with smaller sample size and combined sex-specific LDpred2 may be a good candidate PRS on the sex with larger sample size. However, in real data analysis, there are limited sex-specific GWAS summary statistics, and sex-agnostic LDpred2 gave the best prediction for most of the traits we investigated, with the exception of traits related to WHR, where we observed that combined sex-specific PRSs performed better than the sex-agnostic PRSs.

However, our study only used autosomes to generate polygenic risk scores, ignoring sex chromosomes, particularly the X chromosome. The X-chromosome has two copies in females, but only one in males. In females, one of the two copies may even be silenced, a process known as X-chromosome inactivation (XCI) (Gendrel and Heard, 2011), and the choice of the silent copy is random or biased toward a specific copy (Wang et al., 2014). These distinct characteristics limit the inclusion of X-chromosomes in genome-wide association studies, also making it difficult to include X-chromosomes in PRS estimates. However, increasingly extensive analyses of X-chromosome–inclusive association studies have been developing, indicating that X-chromosome–inclusive PRS may be possible in the future (Chen et al., 2021).

**FIGURE 3:**
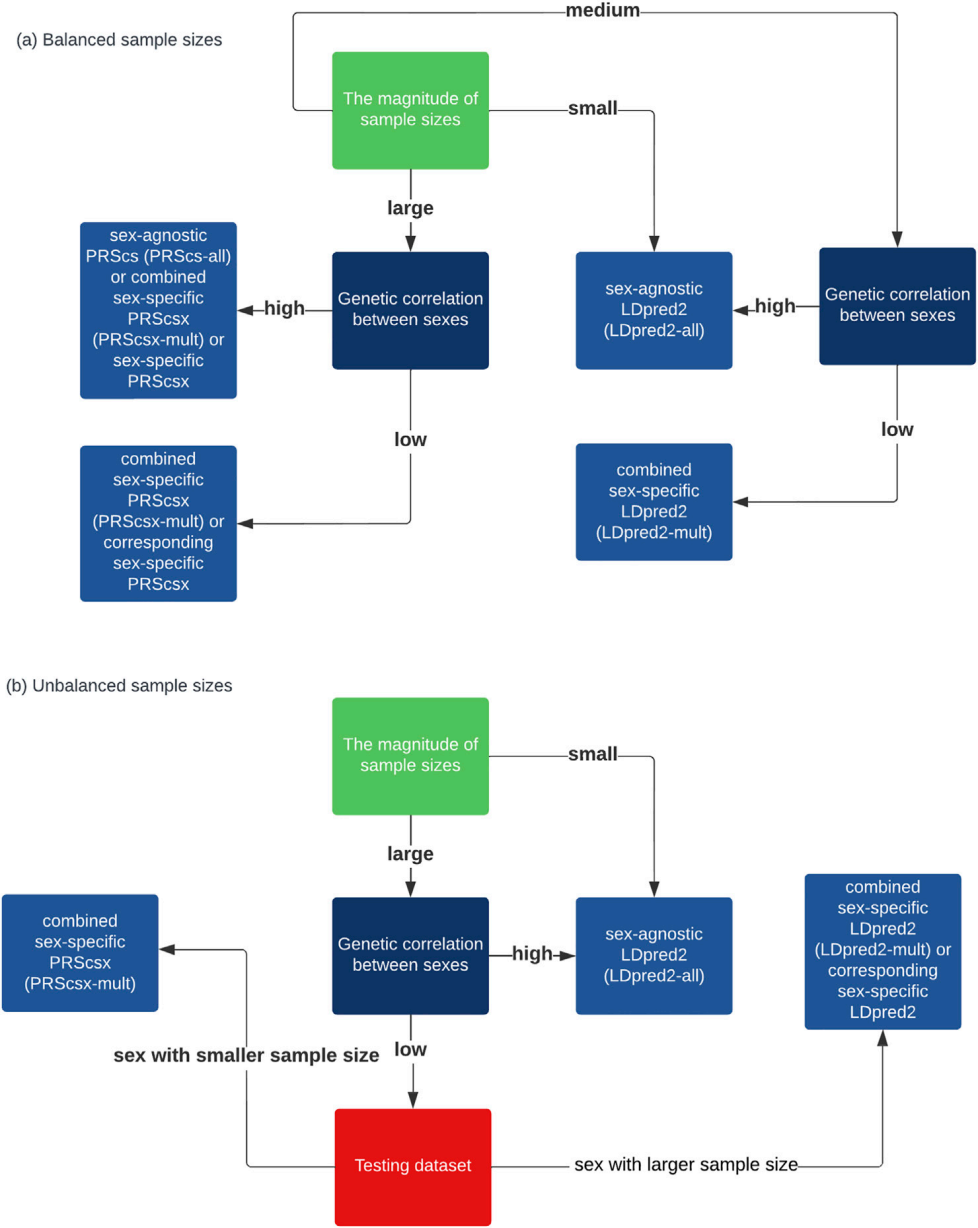
Flow charts that provide a synthesized guide for practitioners to choose among these methods. **(A)** When the sample sizes are balanced between sexes. **(B)** When the sample sizes are unbalanced between sexes.

In conclusion, although there are sex differences in the genetic architecture for some traits, considering these differences, for example, through building models from sex-specific GWAS summary statistics, PRS prediction does not lead to improved prediction accuracy in most traits in our analysis of real data for the time being. Sex-agnostic PRSs remain the best option for most cases. When the sample size is large and the genetic correlation is relatively small between males and females, there may be some benefit to considering sex-specific information for PRS. When there is a big difference in heritability or the sample sizes of the two sexes are very unbalanced, combined sex-specific PRSs are worth considering. A guidance on how to select the optimal PRSs based on simulation results is also shown on Figure 3. However, it is possible that the small sample size limits the use of sex-specific or combined sex-specific PRSs. With larger GWAS sample sizes and more sex-specific GWAS summary statistics becoming available in future studies, PRSs built by considering sex differences and borrowing information from the opposite sex at the same time, known as combined sex-specific PRSs, may be considered in addition to sex-agnostic PRS methods.

## Data Availability

The data analyzed in this study is subject to the following licenses/restrictions: we conducted the research using the individual data from the UK Biobank resource under an approved data request (ref: 29900). This individual data set is not publicly available. We also applied many GWAS data from the GIANT consortium which is publicly accessible (https://portals.broadinstitute.org/collaboration/giant/index.php/GIANT_consortium_data_files#GWAS_Anthropometric_2014_Height_Summary_Statistics). Requests to access UK Biobank individual data should be directed to https://www.ukbiobank.ac.uk/enable-your-research/register.
