# Supplementary material for: Comparison of Methods Utilizing Sex-Specific PRSs Derived From GWAS Summary Statistics
Source: Front Genet. 2022 Jul 8;13:892950. doi: 10.3389/fgene.2022.892950 (PMC9304553; doi:10.3389/fgene.2022.892950)
Supplement: Supplementary file 1 [file DataSheet1.zip › Supplementary Material.docx]

Supplementary Material

# Supplementary Figures


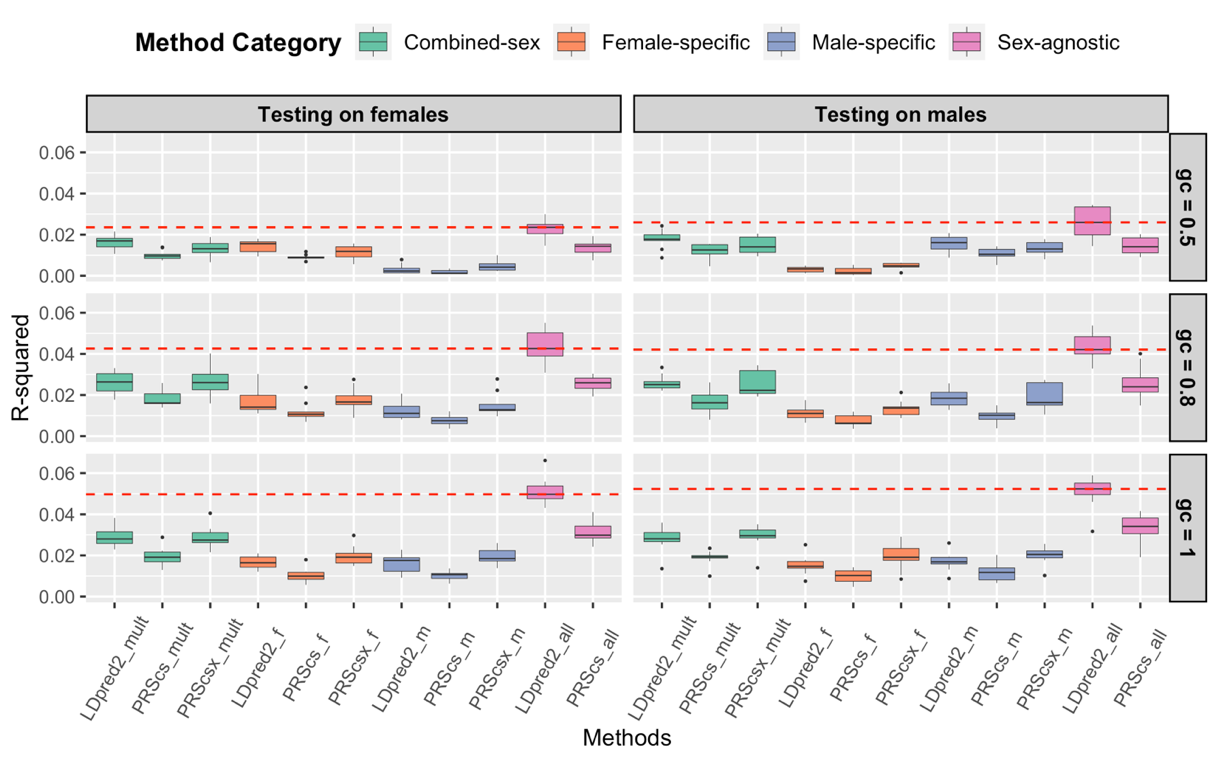


Supplementary Figure 1: Comparisons of PRSs using balanced sample sizes (female/male: 5,000/5,000). The heritability of females is 0.3 and the heritability of males is 0.3. **Female-Specific:** Using female-specific GWAS summary statistics as input. **Male-Specific:** Using male-specific GWAS summary statistics as input. **Sex-agnostic:** Using sex-agnostic GWAS summary statistics as input. **Combined-sex:** The combination of female-specific PRS and male-specific PRS. The estimates from ten replicates are represented by the points in each box. Red dash lines indicate the best performance in each setting.


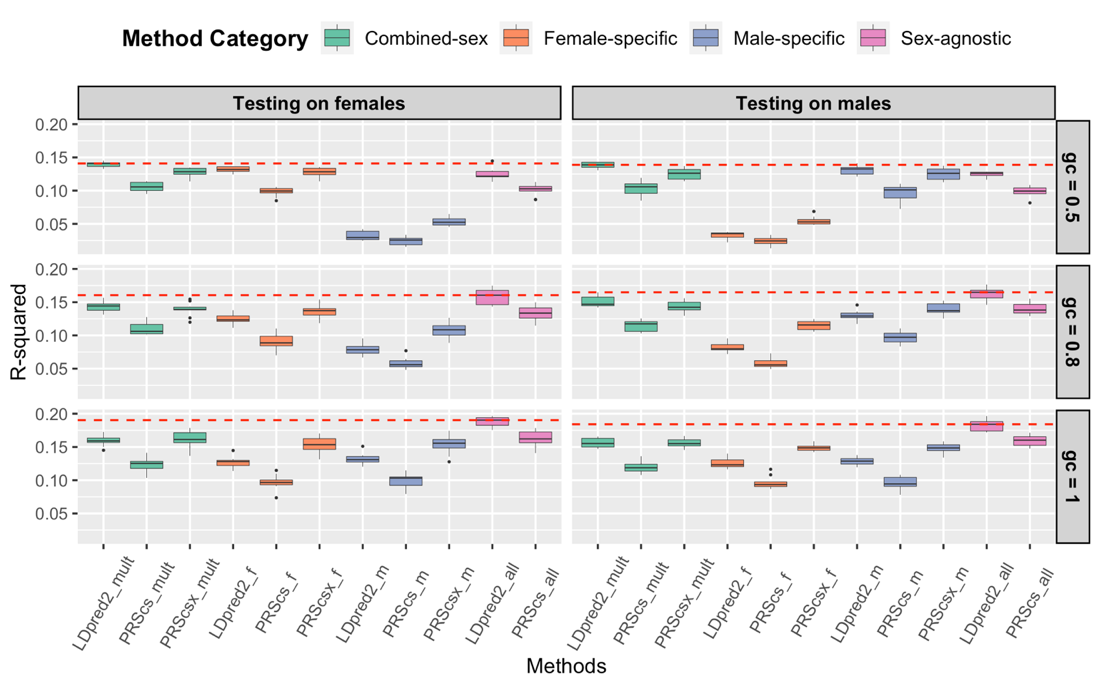


Supplementary Figure 2: Comparisons of PRSs using balanced sample sizes (female/male: 25,000/25,000). The heritability of females is 0.3 and the heritability of males is 0.3. **Female-Specific:** Using female-specific GWAS summary statistics as input. **Male-Specific:** Using male-specific GWAS summary statistics as input. **Sex-agnostic:** Using sex-agnostic GWAS summary statistics as input. **Combined-sex:** The combination of female-specific PRS and male-specific PRS. The estimates from ten replicates are represented by the points in each box. Red dash lines indicate the best performance in each setting.


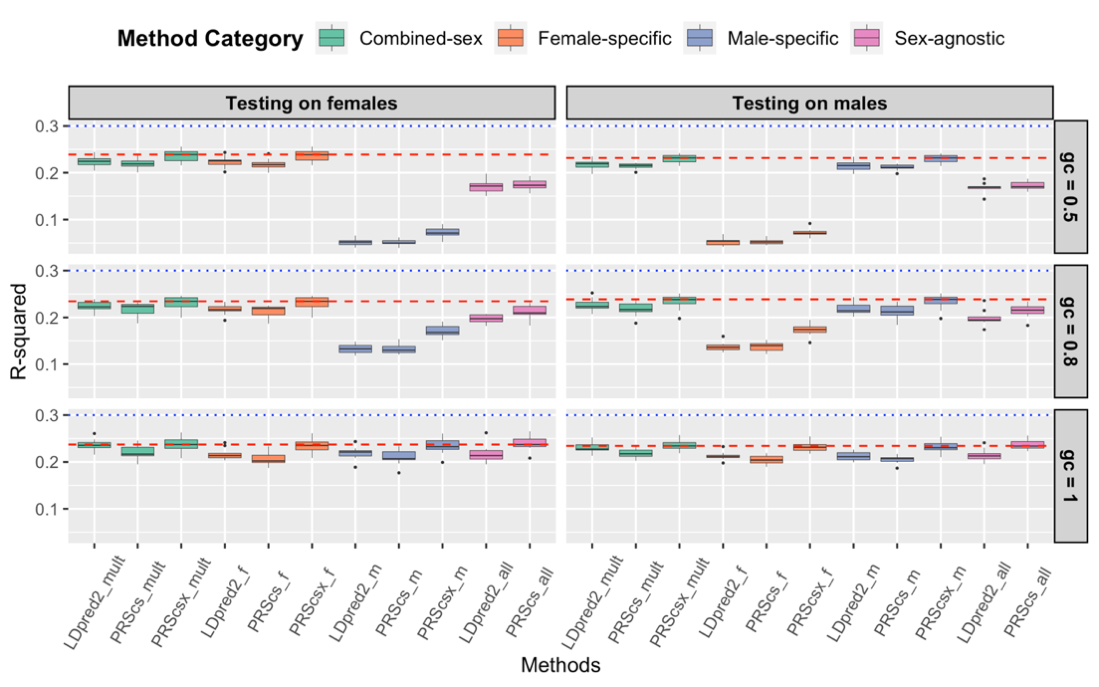


Supplementary Figure 3: Comparisons of PRSs using balanced sample sizes (female/male: 100,000/100,000). The heritability of females is 0.3 and the heritability of males is 0.3. **Female-Specific:** Using female-specific GWAS summary statistics as input. **Male-Specific:** Using male-specific GWAS summary statistics as input. **Sex-agnostic:** Using sex-agnostic GWAS summary statistics as input. **Combined-sex:** The combination of female-specific PRS and male-specific PRS. The estimates from ten replicates are represented by the points in each box. Red dash lines indicate the best performance in each setting. Blue dash lines indicate heritability, the best performance PRS can reach.


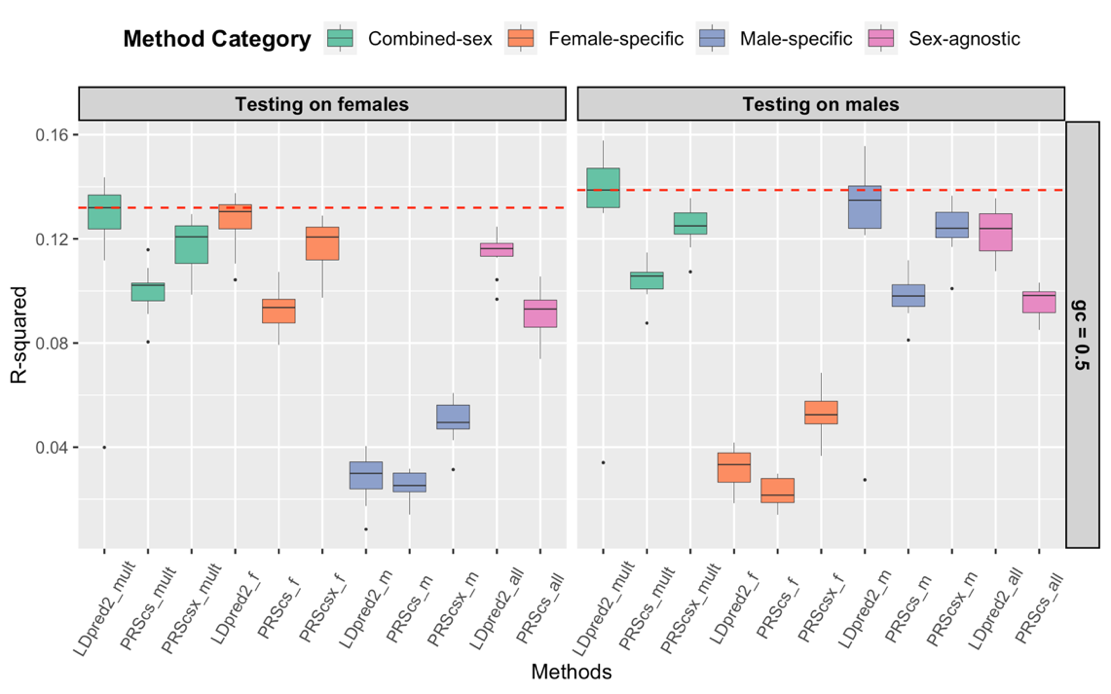


Supplementary Figure 4: Comparisons of PRSs using balanced sample sizes (female/male: 25,000/25,000). The heritability of females is 0.3 and the heritability of males is 0.3. There are 80% causal SNPs shared across sexes. **Female-Specific:** Using female-specific GWAS summary statistics as input. **Male-Specific:** Using male-specific GWAS summary statistics as input. **Sex-agnostic:** Using sex-agnostic GWAS summary statistics as input. **Combined-sex:** The combination of female-specific PRS and male-specific PRS. The estimates from ten replicates are represented by the points in each box. Red dash lines indicate the best performance in each setting.


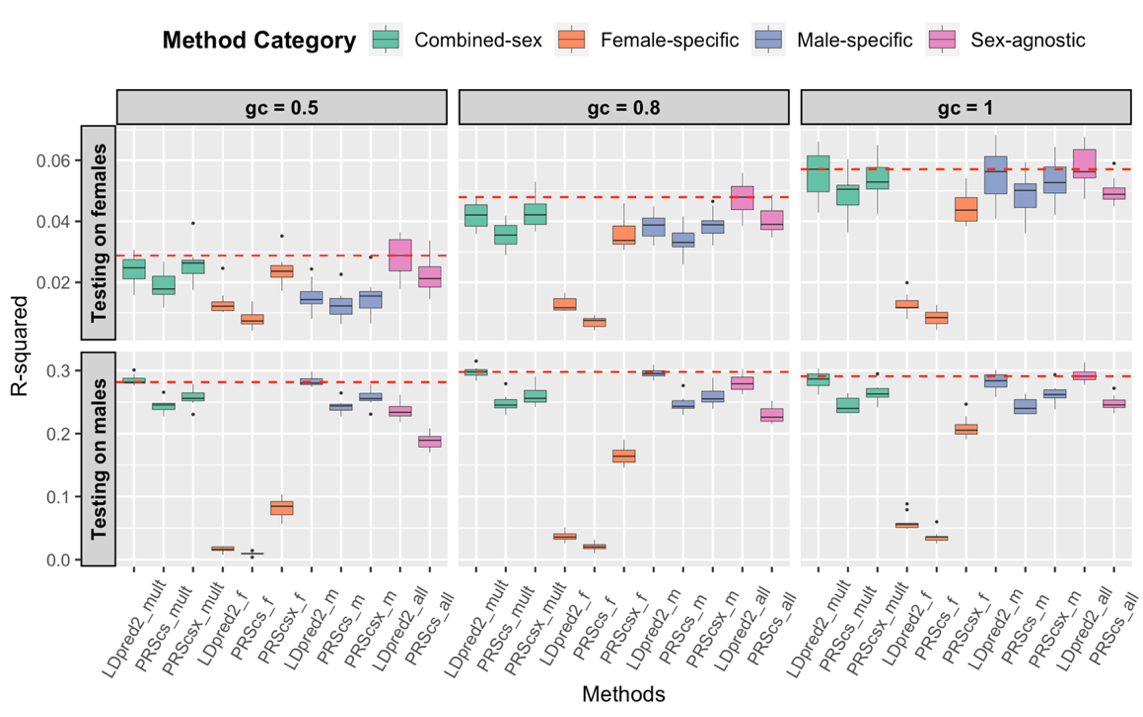


Supplementary Figure 5: Comparisons of PRSs using balanced sample sizes (female/male: 25,000/25,000). The heritability of females is 0.1 and the heritability of males is 0.5. **Female-Specific:** Using female-specific GWAS summary statistics as input. **Male-Specific:** Using male-specific GWAS summary statistics as input. **Sex-agnostic:** Using sex-agnostic GWAS summary statistics as input. **Combined-sex:** The combination of female-specific PRS and male-specific PRS. The estimates from ten replicates are represented by the points in each box. Red dash lines indicate the best performance in each setting.


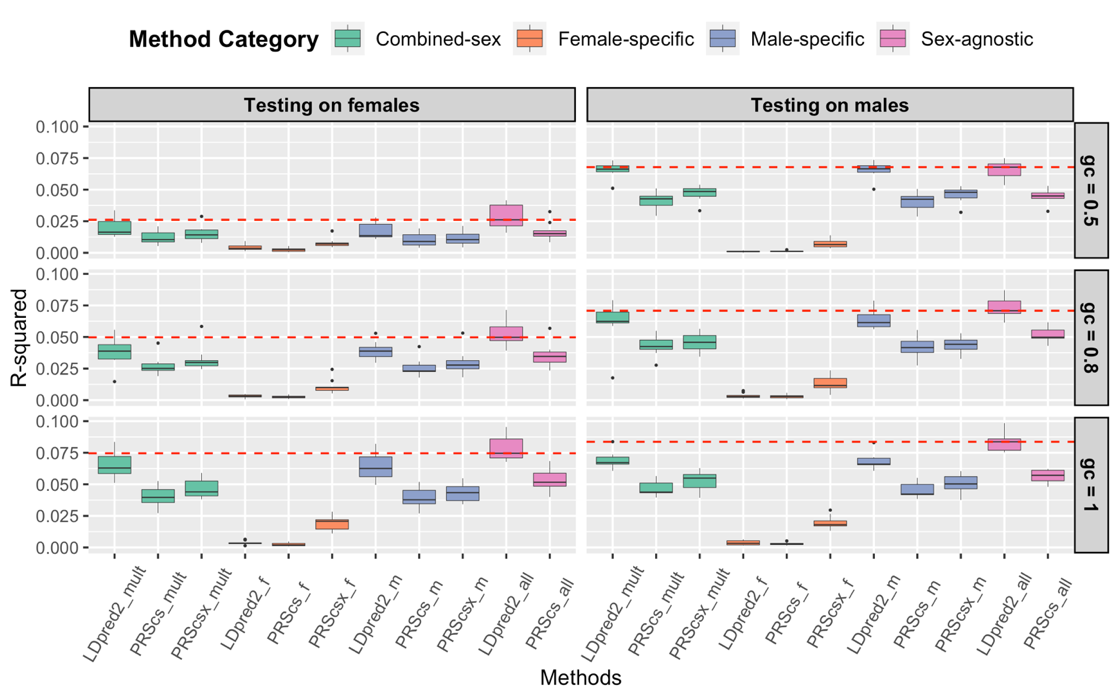


Supplementary Figure 6: Comparisons of PRSs using unbalanced sample sizes (female/male: 2,500/12,500). The heritability of females is 0.3 and the heritability of males is also 0.3. **Female-Specific:** Using female-specific GWAS summary statistics as input. **Male-Specific:** Using male-specific GWAS summary statistics as input. **Sex-agnostic:** Using sex-agnostic GWAS summary statistics as input. **Combined-sex:** The combination of female-specific PRS and male-specific PRS. The estimates from ten replicates are represented by the points in each box.


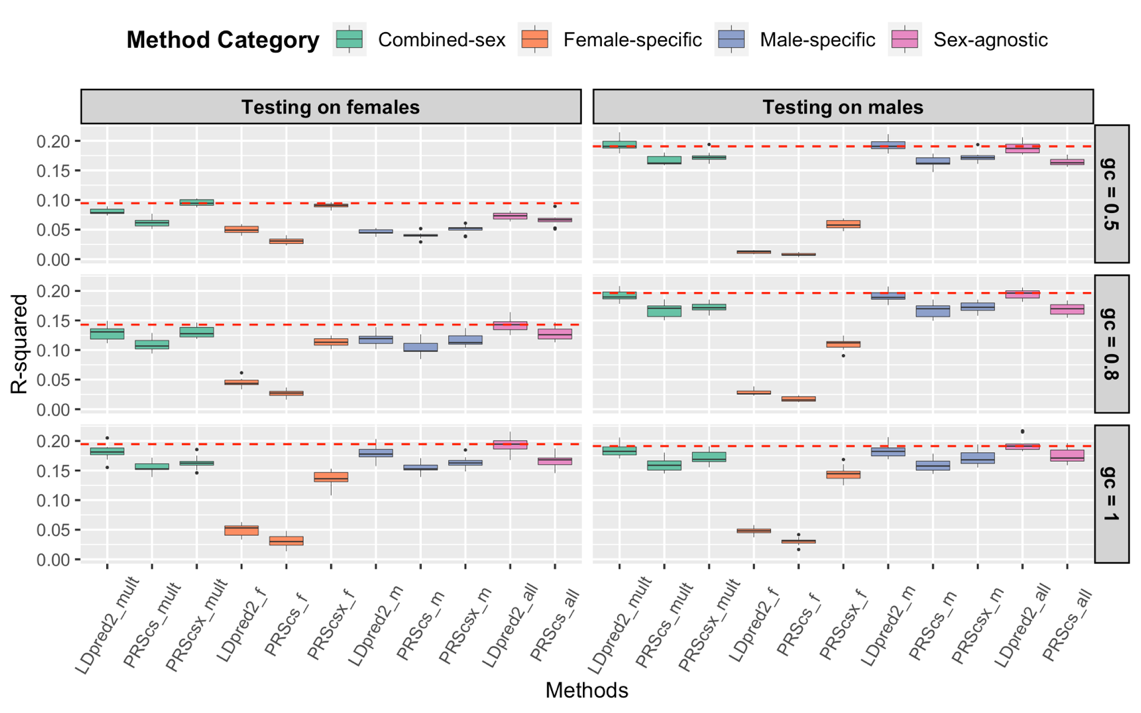


Supplementary Figure 7: Comparisons of PRSs using unbalanced sample sizes (female/male: 10,000/50,000). The heritability of females is 0.3 and the heritability of males is also 0.3. **Female-Specific:** Using female-specific GWAS summary statistics as input. **Male-Specific:** Using male-specific GWAS summary statistics as input. **Sex-agnostic:** Using sex-agnostic GWAS summary statistics as input. **Combined-sex:** The combination of female-specific PRS and male-specific PRS. The estimates from ten replicates are represented by the points in each box.


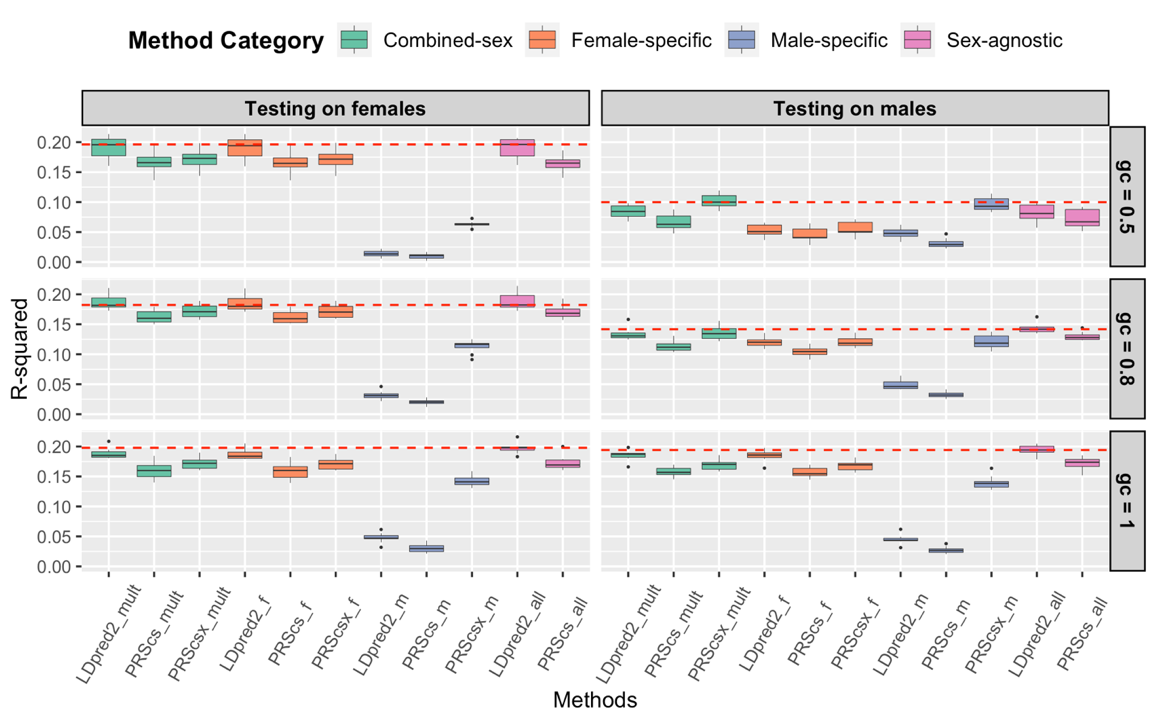


Supplementary Figure 8: Comparisons of PRSs using unbalanced sample sizes (female/male: 50,000/10,000). The heritability of females is 0.3 and the heritability of males is also 0.3. **Female-Specific:** Using female-specific GWAS summary statistics as input. **Male-Specific:** Using male-specific GWAS summary statistics as input. **Sex-agnostic:** Using sex-agnostic GWAS summary statistics as input. **Combined-sex:** The combination of female-specific PRS and male-specific PRS. The estimates from ten replicates are represented by the points in each box.

#####
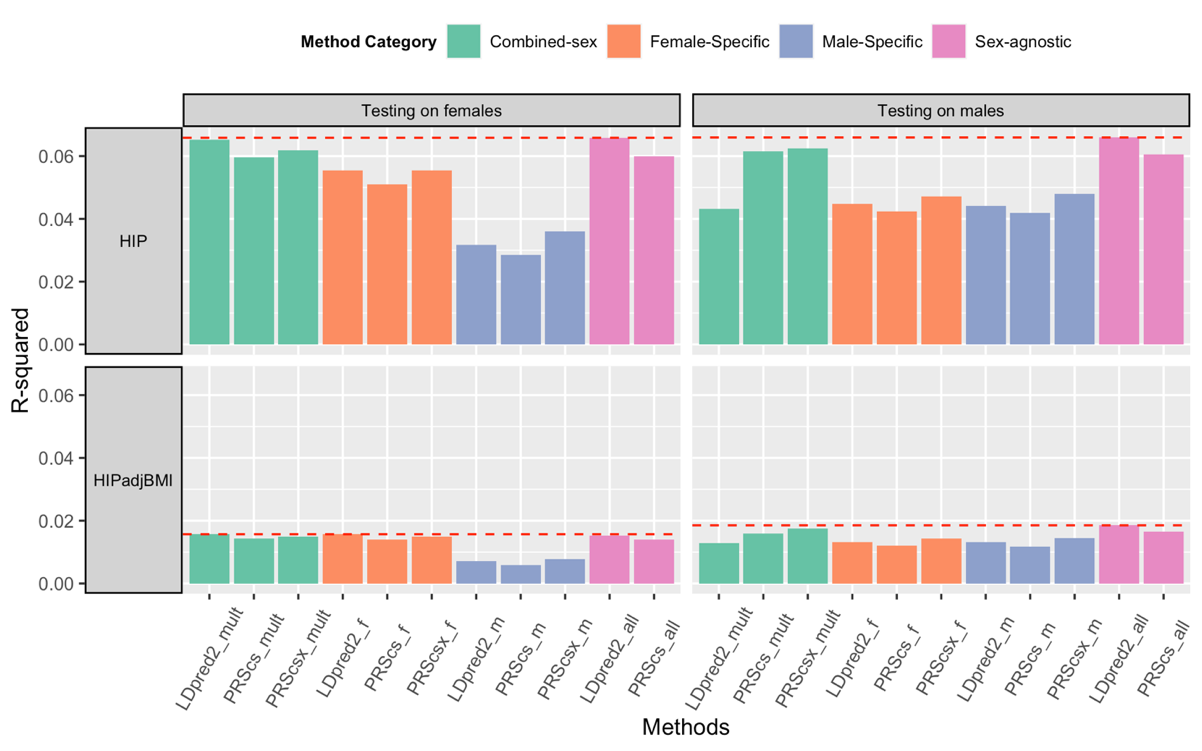


Supplementary Figure 9: Comparisons of PRSs on HIP, HIPadjBMI. (HIP:hip circumference; HIPadjBMI: HIP adjusted by BMI). **Female-Specific:** Using female-specific GWAS summary statistics as input. **Male-Specific:** Using male-specific GWAS summary statistics as input. **Sex-agnostic:** Using sex-agnostic GWAS summary statistics as input. **Combined-sex:** The combination of female-specific PRS and male-specific PRS. The red dash lines indicate the best performances.


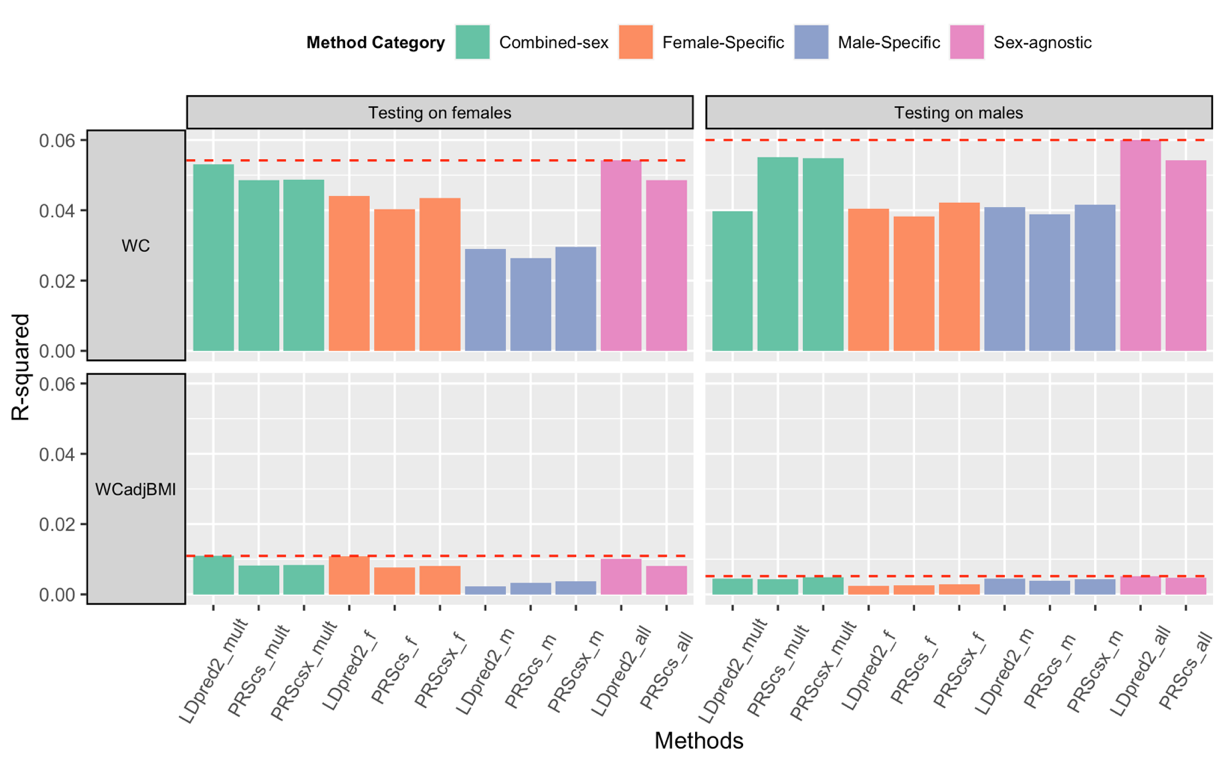


Supplementary Figure 10: Comparison of PRSs on WC, WCadjBMI. (WC: waist circumference; WCadjBMI: WC adjusted by BMI). **Female-Specific:** Using female-specific GWAS summary statistics as input. **Male-Specific:** Using male-specific GWAS summary statistics as input. **Sex-agnostic:** Using sex-agnostic GWAS summary statistics as input. **Combined-sex:** The combination of female-specific PRS and male-specific PRS. The red dash lines indicate the best performances.


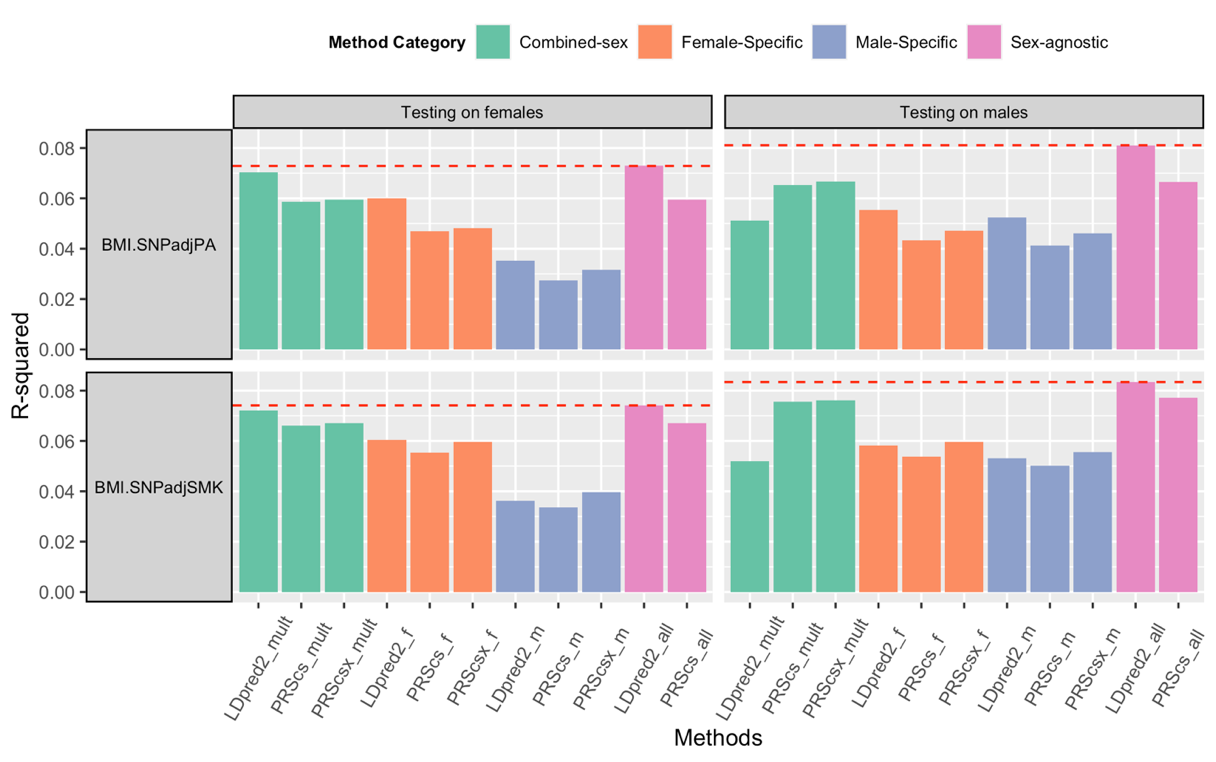


Supplementary Figure 11: Comparisons of PRSs on BMI.SNPadjPA and BMI.SNPadjSMK. (BMI.SNPadjPA: with physical activity level as a covariate; BMI.SNPadjSMK: with smoking status as a covariate). **Female-Specific:** Using female-specific GWAS summary statistics as input. **Male-Specific:** Using male-specific GWAS summary statistics as input. **Sex-agnostic:** Using sex-agnostic GWAS summary statistics as input. **Combined-sex:** The combination of female-specific PRS and male-specific PRS. The red dash lines indicate the best performances.


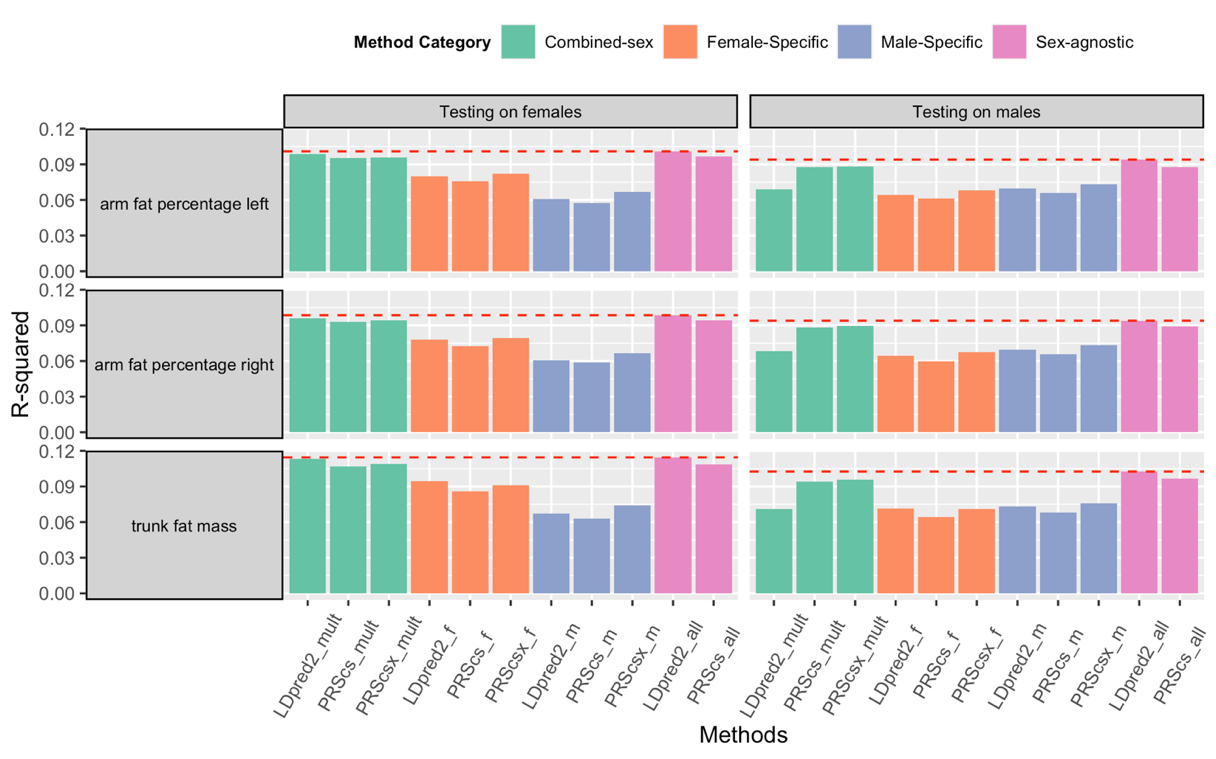


Supplementary Figure 12: Comparisons of PRSs on arm fat percentage (left/right) and trunk fat mass. **Female-Specific:** Using female-specific GWAS summary statistics as input. **Male-Specific:** Using male-specific GWAS summary statistics as input. **Sex-agnostic:** Using sex-agnostic GWAS summary statistics as input. **Combined-sex:** The combination of female-specific PRS and male-specific PRS. The red dash lines indicate the best performances.


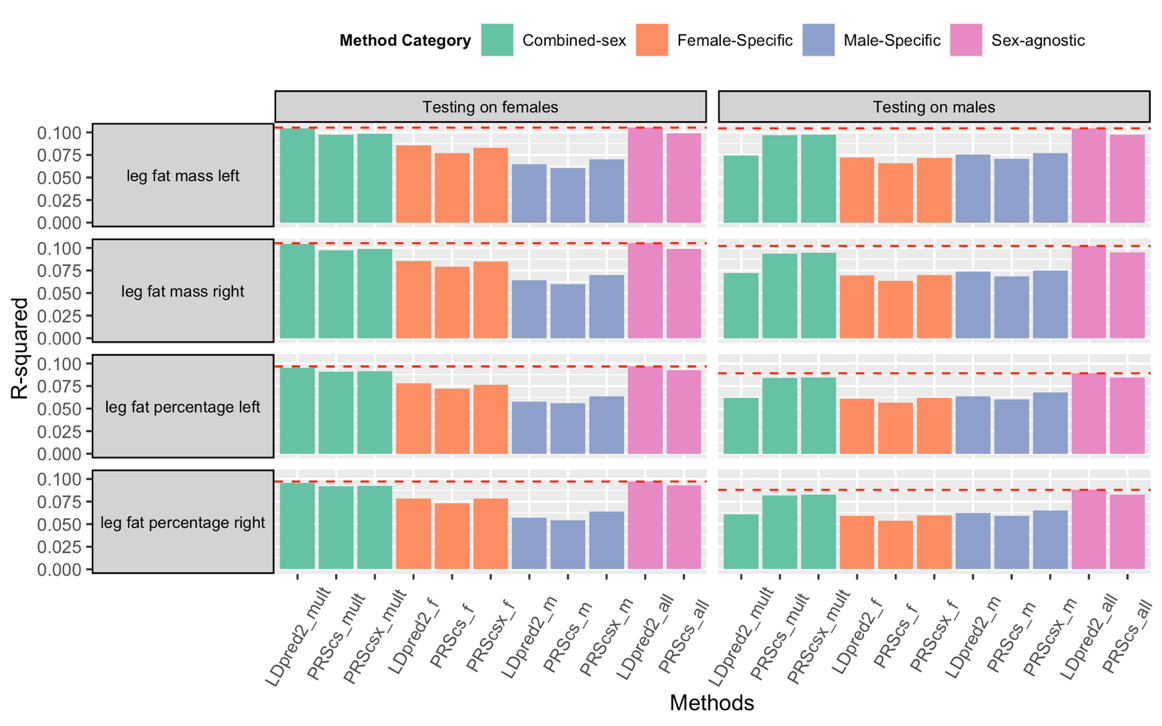


Supplementary Figure 13: Comparisons of PRSs on leg fat mass (left/right) and leg fat percentage (left/right). **Female-Specific:** Using female-specific GWAS summary statistics as input. **Male-Specific:** Using male-specific GWAS summary statistics as input. **Sex-agnostic:** Using sex-agnostic GWAS summary statistics as input. **Combined-sex:** The combination of female-specific PRS and male-specific PRS. The red dash lines indicate the best performances.

# Supplementary Tables

| **Traits** | **Male Sample Size** | **Female Sample Size** | **Male Heritability** **(SE)** | **Female Heritability**  **(SE)** | **Genetic Correlation**  **(SE)** |
| --- | --- | --- | --- | --- | --- |
| **WHR** | 57571 | 85548 | 0.121 (0.011) | 0.101 (0.011) | 0.708 (0.021) |
| **WHRadjBMI** | 56976 | 84305 | 0.109 (0.094) | 0.094 (0.014) | 0.664 (0.028) |
| **HIP** | 57858 | 86057 | 0.161 (0.013) | 0.165 (0.010) | 0.784 (0.017) |
| **HIPadjBMI** | 57360 | 84870 | 0.185 (0.016) | 0.143 (0.013) | 0.797 (0.014) |
| **WC** | 61776 | 90645 | 0.155 (0.012) | 0.152 (0.009) | 0.800 (0.018) |
| **WCadjBMI** | 61424 | 89861 | 0.155 (0.013) | 0.103 (0.010) | 0.738 (0.019) |
| **BMI.SNPadjPA** | 51928 | 79877 | 0.179 (0.013) | 0.165 (0.009) | 0.780 (0.013) |
| **WHRadjBMI.SNPadjPA** | 43623 | 65902 | 0.110 (0.012) | 0.115 (0.013) | 0.644 (0.024) |
| **WCadjBMI.SNPadjPA** | 49775 | 73927 | 0.173 (0.012) | 0.110 (0.009) | 0.736 (0.017) |
| **BMI.SNPadjSMK** | 63656 | 92966 | 0.157 (0.013) | 0.149 (0.009) | 0.789 (0.015) |
| **WHRadjBMI.SNPadjSMK** | 48534 | 73086 | 0.089 (0.012) | 0.092 (0.012) | 0.660 (0.031) |
| **WCadjBMI.SNPadjSMK** | 55063 | 79925 | 0.170 (0.013) | 0.111 (0.010) | 0.727 (0.018) |

Supplementary Table 1: Characteristics of GWAS data from GIANT: sample sizes of each sex; heritability (SE) for both sexes; genetic correlation (SE) between sexes. (WHR: waist and hip ratio; WHRadjBMI: WHR adjusted by BMI; HIP: hip circumference ; HIPadjBMI : HIP adjusted by BMI ; WC: waist circumference; WCadjBMI: WC adjusted by BMI ; BMI.SNPadjPA : added covariate of physical activity level; WHRdjBMI.SNPadjPA: added covariate of physical activity level; WCadjBMI.SNPadjPA: added covariate of physical activity level ; BMI.SNPadjSMK; added covariate of smoking status; WHRadjBMI.SNPadjSMK: added covariate of smoking status; WCadjBMI.SNPadjSMK: added covariate of smoking status

| **Traits** | **Male Sample Size** | **Female Sample Size** | **Male Heritability** **(SE)** | **Female Heritability** **(SE)** | **Genetic Correlation (SE)** |
| --- | --- | --- | --- | --- | --- |
| **Arm fat percentage left** | 100,007 | 119,717 | 0.240 (0.010) | 0.263 (0.010) | 0.784 (0.008) |
| **Arm fat percentage right** | 100,021 | 119,741 | 0.243 (0.010) | 0.255 (0.010) | 0.785 (0.008) |
| **Leg fat percentage left** | 100,030 | 119,749 | 0.235 (0.010) | 0.254 (0.010) | 0.788 (0.008) |
| **Leg fat percentage right** | 100,042 | 119,754 | 0.230 (0.010) | 0.255 (0.010) | 0.791 (0.008) |
| **Leg fat mass left** | 100,030 | 119,748 | 0.259 (0.011) | 0.271 (0.011) | 0.796 (0.008) |
| **Leg fat mass right** | 100,037 | 119,753 | 0.252 (0.011) | 0.271 (0.011) | 0.795 (0.008) |
| **Trunk fat mass** | 99,978 | 119,689 | 0.250 (0.010) | 0.278 (0.011) | 0.790 (0.007) |

Supplementary Table 2: Characteristics of GWAS data from the UK Biobank: sample sizes of each sex; heritability for both sexes; genetic correlation between sexes.
